# USE OF AND EXPERIENCES WITH TELEHEALTH VISITS AND PATIENT PORTALS IN A NATIONALLY REPRESENTATIVE SAMPLE OF OLDER ADULTS

**DOI:** 10.1093/geroni/igae098.0084

**Published:** 2024-12-31

**Authors:** Erica Solway, Dianne Singer, Matthias Kirch, Scott Roberts, Jeff Kullgren

**Affiliations:** University of Michigan, Ann Arbor, Michigan, United States; University of Michigan, Ann Arbor, Michigan, United States; University of Michigan, Ann Arbor, Michigan, United States; University of Michigan, Ann Arbor, Michigan, United States; University of Michigan, Ann Arbor, Michigan, United States

## Abstract

The University of Michigan National Poll on Healthy Aging (NPHA) has explored older adults’ changing views and experiences related to telehealth and other health-related technologies over the past several years. NPHA surveys are fielded twice per year to a nationally representative sample of over 2,500 adults ages 50-80 (completion rates: 50-60%). When the poll first included questions about telehealth use and perceptions in 2019, just 4% of U.S. adults ages 50-80 reported having a telehealth visit in the past year. Telehealth expanded rapidly during the pandemic and by mid-2020, 26% of older adults reported having had a telehealth visit within the prior three months. In addition, the NPHA has explored patient portal use, documenting an increase in portal uptake over time as well as older adults’ experiences with, and barriers to, portal use. Recently, the NPHA explored older adults’ experiences related to sending portal messages to their health care providers and the out-of-pocket costs associated with this. Other recent NPHA research has demonstrated use and interest in direct-to-consumer telehealth services provided by online-only companies. As of 2023, the poll found that 7.5% of people ages 50-80 had used at least one direct-to-consumer health care service from an online-only provider, and 32% said they would be interested in these types of health care services in the future. The NPHA provides timely insights on older adults’ changing perspectives and experiences related to emerging areas of health care that can help to inform efforts to improve access, quality, and integration of health care.

